# Pleural Epithelioid Hemangioendothelioma: Literature Summary and Novel Case Report

**DOI:** 10.14740/jocmr2174w

**Published:** 2015-05-08

**Authors:** Julita Salijevska, Robert Watson, Amy Clifford, Andrew I. Ritchie, Francisco Mauri, David Adeboyeku

**Affiliations:** a Imperial College Healthcare NHS Trust, Charing Cross Hospital, Fulham Palace Road, London W6 8RF, UK; b The North West London Hospitals NHS Trust, Northwick Park Hospital, Watford Road, Harrow, Middlesex, HA1 3UJ, UK; c Imperial College Healthcare NHS Trust, The Hammersmith Hospital, Du Cane Road, London W12 0HS, UK

**Keywords:** Epithelioid hemangioendothelioma, Pleura, Pulmonary hemangioendothelioma, Intravascular bronchoalveolar tumor

## Abstract

Epithelioid hemangioendothelioma (EHE) is a rare malignant cancer of vascular origin that can affect multiple and varied tissue sites. A subtype of EHE, pulmonary epithelioid hemangioendothelioma (PHE), is more unusual with only 200 reported cases. Of these, only 27 have been classified as pleural in origin. Based on available literature, the average age of presentation of pleural PHE is 45.7 years with a male preponderance of 2.375. A summary of all published case reports reveals significant heterogeneity both in presentation and management. Here we add to this knowledge-base with a report of an unusual case of pleural PHE in a 36-year-old female who presented with a 6-week history of chest pain and breathlessness. Significant challenges in the diagnosis and management of patients with pleural PHE exist, including a wide initial differential diagnosis and difficulties in obtaining tissue specimens, coupled with relatively limited treatment options. Early referral to a cardiothoracic center for video-assisted thoracoscopic biopsy is crucial in facilitating a diagnosis and allowing adequate pleural drainage for symptomatic relief.

## Introduction

Epithelioid hemangioendothelioma (EHE) is a rare malignant cancer of vascular origin. Cases have been reported affecting bone, brain, the meninges, soft tissue, gastrointestinal tract, mediastinum, spleen, breast, testicle and skin with a variable grade of malignancy [1, 2]. It typically follows an intermediate course between hemangioma and conventional angiosarcoma [3-5]; however, the pleural variant of the disease is less frequently described and is known to follow a more aggressive clinical course [2].

Pulmonary epithelioid hemangioendothelioma (PHE) was first described in 1975 by Dail and Leibow [2]. However, due to its low incidence, the true epidemiology of PHE remains unknown. To our knowledge, there have been less than 200 cases of PHE reported in the literature with only 27 cases further classified as pleural epithelioid hemangioendothelioma, as shown in Table 1 [1, 6-21].

**Table 1:** Summary of Published Cases of Pleural PHE

Authors	Age/gender	Symptom at presentation	Imaging	Other sites involved	Treatment	Reported survival (months)
Lee et al, 2008 [1]	31/F	Chest pain	Pleural thickening on CT	Lung, bone	Adriamycin, MAID	10
Pinet, 1999 [6]	50/F	NA	Right pleural effusion	Ascites	Carboplatin and etoposide	18+
Crotty et al, 2000 [7]	51 - 71/M n = 4	Chest pain (3), dyspnea (3), cough, fever (1), weight loss (1)	Pleural effusions, nodules and thickening	Lung (2), LN, liver and retroperitoneum (1)	Unknown	1 - 19 (10 on average)
Lazarus, 2011 [8]	42/M +42/M n = 2	1. Cough, dyspnea, chest pain 2. Fever, cough	1. Right pleural effusion 2. Left pleural effusion	1. Skin 2. No	1. Taxol and bevacizumab 2. Carboplatin, etoposide, and bevacizumab	8 6
Yousem and Hochholzer, 1987 [9]	34/M	Dyspnea	Bilateral pleural effusions	No	None	3
Lin, 1996 [10]	36 - 58/M n = 6	NA	Pleural effusion (n = 6)	Pericardial effusion n = 1	NA	NA
Al-Sharim et al, 2005 [11]	51/M	Cough, dyspnea	Left pleural effusion	Skin	INF-alpha	24+
Vitorio, 2004 [12]	61/M	Chest pain	Left pleural effusion and thickening	No	Cisplatin and etoposide	3
Saqi, 2007 [13]	37/M	Dyspnea, chest pain	Right pleural effusion	No	NA	NA
Liu et al, 2010 [14]	80/M	Dyspnea	Right-sided effusion	No	Surgical, chemotherapy unknown	6
Bocchino, 2010 [15]	58/F	Cough, dyspnea, chest pain	Pleural mass	No	None	3
Andre, 2010 [16]	65/F	Chest pain	Pleural effusion		Carboplatin, etoposide	6
Kim et al, 2011 [17]	46/F	Cough, chest discomfort	Right pleural effusion	No	Surgical, carboplatin, etoposide	22+
Marquez-Medina, 2011 [18]	85/M	Chest and shoulder pain, fatigue, weight loss	Pleural effusion		None	7
Bansal, 2012 [19]	51/F	Chest pain, weight loss	Pleural effusion and thickening	No	Doxorubicin	4
Yu, 2013 [20]	39/F	Dyspnea	Pleural mass on CT	Myocardial compression	Surgical, carboplatin, etoposide	14+
Ha, 2014 [21]	71/M	Cough, dyspnea, fatigue	Bilateral pleural effusions	Pericardial effusion, lung	NA	NA
This case, 2014	36/F	Chest pain	Right whiteout	Lungs	Paclitaxel	6

Analysis of these cases reveals significant heterogeneity in both presentation and management (Table 1). The mean age at presentation is 45.7 years with a female/male ratio of 1:2.375 and a mean survival of 9.6 months. There was a heterogenous array of symptoms at presentation; however, chest pain was a consistent feature, along with dyspnea and cough. No consistent management approach was used with some patients undergoing surgery, some receiving chemotherapy, some a combination and some no specific treatment. Of the patients that received chemotherapy a variety of regimens were used; however, carboplatin and etoposide was the most popular with six out of 11 patients receiving this combination of treatment.

Here we describe a more unusual case of pleural PHE in a young female presenting with a 6-week history of right-sided chest pain, found to have a complete whiteout on chest radiograph secondary to a malignant pleural effusion. This adds further to the limited published knowledge of pleural PHE and gives further insight into the investigation and management options.

## Case Report

A 36-year-old Caucasian female attended the accident and emergency department with right-sided chest pain. The pain was described as dull and aching, with a pleuritic nature. It was sited diffusely over the right anterior and lateral chest wall and was reproducible on palpation. The pain had increased in severity over the preceding 6 weeks and at the time of admission was associated with mild dyspnea. Prior to presentation, the patient reported no weight loss, fevers or night sweats. Her family history and past medical history were unremarkable. However, she was a cigarette smoker (10 pack-year history).

On examination, the patient was pale and tachypnoeic. Her observations demonstrated a borderline tachycardia at 92 beats per minute and pulse oximetry of 92% on room air. On examination of the respiratory system, there was decreased expansion, dullness to percussion and decreased breath sounds over the right hemithorax. There was no jaundice, digital clubbing, skin changes or palpable lymphadenopathy. The breast examination was unremarkable.

Initial laboratory findings demonstrated a leukocytosis (white blood cells 18.6 × 10^9^/L) and neutrophilia (neutrophils 15.9 × 10^9^/L), anemia (hemoglobin 9.8 g/dL), thrombocytosis (platelets 518 × 10^9^/L), hypercalcemia (corrected calcium 2.81 mmol/L), and raised C-reactive protein (117 mg/L).

**Figure 1:**
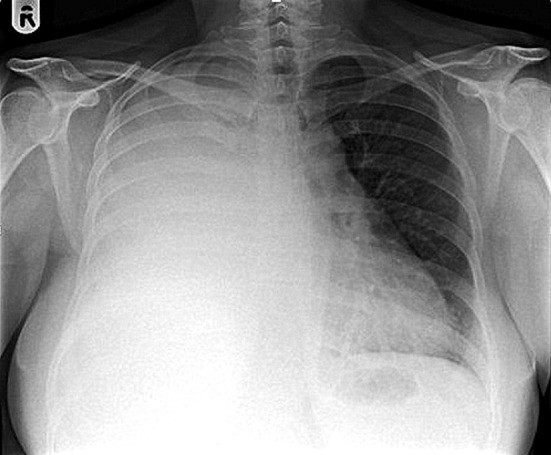
Chest radiograph demonstrating complete opacification of the right hemithorax with mild mediastinal shift to the left side.

**Figure 2:**
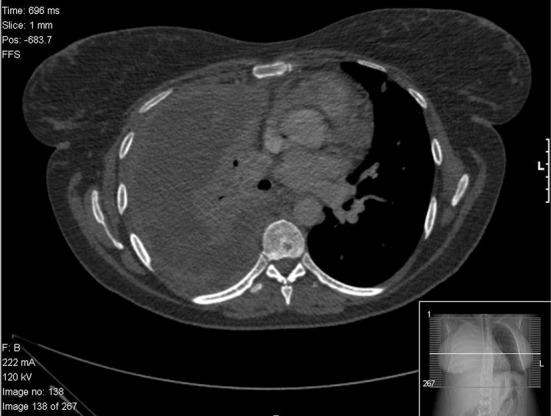
CT scan demonstrating pleural thickening, effusion, right lung collapse and left mediastinal shift.

A chest radiograph revealed complete opacification of the right hemithorax with mild mediastinal shift to the left side (Fig. 1). This was further characterized by a contrast enhanced computed tomography (CT) scan of the thorax, abdomen and pelvis which showed a large loculated right pleural effusion with associated right lung collapse, significant multifocal right-sided pleural thickening, and multiple pulmonary nodules on the left. No abnormalities were seen below the diaphragm, save for some simple renal cysts. Appearances were reported to be likely in keeping with pleural and pulmonary metastases (Fig. 2).

The patient went on to have an ultrasound guided Seldinger chest drain inserted. Seven hundred twenty milliliters of heavily blood stained pleural fluid drained and was sent for analysis: pH 6.99, protein 52 g/L, and LDH 642 IU/L. Cytological analysis did not detect any malignant cells. The microscopy and subsequent culturing was unremarkable.

Considering the patient’s young age and the radiological findings, our provisional diagnosis was malignancy with a likely primary of breast, ovary, lymphoma or lung. With this in mind our patient went on to have an ultrasound examination of her breasts, which revealed some areas of benign breast changes but no focal suspicious appearances. Transvaginal ultrasound demonstrated an anteverted uterus, endometrial thickness of 5 mm and normal ovaries.

Despite chest drain insertion and the initial volume drained, we were subsequently unable to remove a significant volume of pleural fluid from the right hemi-thorax. The patient was referred to a tertiary center with cardiothoracic facilities where video-assisted thoracoscopic surgery (VATS) was performed. This allowed successful evacuation of the septated pleural effusion and the opportunity to perform pleural biopsies.

**Figure 3:**
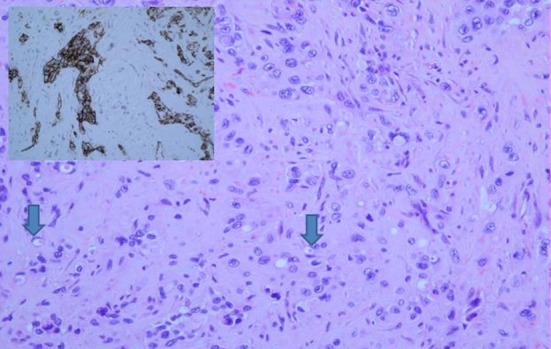
Histology from VATS guided pleural biopsy. Tumor shows cords, nests and groups of medium or large sized epithelioid cells with inconspicuous nucleoli set in a myxoid stroma. Some cells show mild atypia. Many cells have intracytoplasmic lumina which occasionally contain red blood cells (arrows). Tumor cells are positive for CD31 (insert) and CD34.

The histological diagnosis was confirmed by both conventional examination demonstrating the characteristic tumor cells with cords and nests, and immunohistochemistry with expression of MNF116 and CK7, D2-40, CD31 and CD34 antibodies (Fig. 3).

Post-operatively our patient recovered well and was referred for specialist oncological input which she received at a quaternary London cancer center. Although an effective treatment for pleural PHE has yet to be established, Pinet et al [6] have reported the case of an aggressive pleural PHE which had a complete response to treatment with carboplatin and etoposide. Our patient received weekly cycles of paclitaxel, a mitotic inhibitor, which has also demonstrated a clinical response in case reports [22], for a 3-month period. Unfortunately, during this time she required frequent admissions for pain control and sadly passed away 6 months after her diagnosis.

## Discussion

Pleural EHE is an extremely rare entity. Detecting it in the early stages is often not possible, as initial symptoms are typically non-specific with chest pain, dyspnea or persistent cough, the most frequently reported presenting complaint [7, 8]. Furthermore literature review has highlighted individual cases of incidental diagnosis in asymptomatic patients [23].

Our case highlights the aggressive nature and late presentation of this malignancy even in a young patient with good pre-morbid baseline functioning. It also demonstrates the need for a systematic approach to all patients presenting with a unilateral pleural effusion of likely malignant origin as more common etiologies need to be excluded prior to referral to a specialist oncology center. In such scenarios, the clinical team should move quickly to establish a diagnosis so that treatment can be started as soon as possible, especially given the poor life expectancy associated with this cancer.

It is worth bearing in mind that pleural PHE can lead to heavily loculated effusions. Therefore VATS can play a vital role in securing a tissue diagnosis and evacuating the pleural effusion when less invasive methods fail.

As with our patient, pleuritic pain is a dominant feature of pleural PHE. Early referral to an acute pain service must be considered in patients that do not respond to conventional analgesia [24] as poorly controlled symptoms may lead to a delay in oncology referral and importantly a delay in starting chemotherapy and radiotherapy.

Further research and case reporting are required to contribute to the data regarding the clinical course and the natural history of this rare malignancy in order to build an evidence base for treatment. A successful and accepted chemotherapeutic treatment regime is yet to be established and adopted with a range of different options currently employed.

### Conclusion

Whilst EHE is an extremely rare malignant cancer, and its pleural subtype is even rarer, it is important we continue to characterize its clinical course and response to treatments. Early cardiothoracic input to allow for adequate pleural drainage and biopsy along with aggressive pain management are important components of the initial management. A standard chemotherapeutic regime is yet to be widely adopted and further work in this area is needed.
